# Intravenous thrombolysis for bioprosthetic valve thrombosis

**DOI:** 10.1093/eurheartj/ehad303

**Published:** 2023-06-02

**Authors:** Rik Adrichem, Ricardo P J Budde, Nicolas M Van Mieghem

**Affiliations:** Department of Interventional Cardiology, Thoraxcenter, Erasmus University Medical Center, Dr Molewaterplein 40, 3015 GD Rotterdam, South-Holland, The Netherlands; Department of Radiology & Nuclear Medicine, Erasmus University Medical Center, Dr Molewaterplein 40, 3015 GD Rotterdam, South-Holland, The Netherlands; Department of Interventional Cardiology, Thoraxcenter, Erasmus University Medical Center, Dr Molewaterplein 40, 3015 GD Rotterdam, South-Holland, The Netherlands

A 77-year-old woman experienced new-onset dyspnoea on exertion 1 year after alcohol septal ablation and transcatheter aortic valve implantation (TAVI) with a balloon-expandable valve for symptomatic severe aortic stenosis and basal septal hypertrophy.

Transthoracic echocardiographic assessment (TTE) revealed an incremental transvalvular peak velocity from 2.9 m/s 3 days after TAVI to 4.3 m/s. Multislice computed tomography (MSCT) demonstrated hypoattenuated leaflet thickening (HALT) of all leaflets and reduced leaflet motion (RLM) of one leaflet (*Panel A*). Her antithrombotic regimen was changed from apixaban 5 mg bi-daily to acenocoumarol with a target international normalized ratio (INR) of 2.5–3.5. Six weeks later, symptoms persisted and the transvalvular peak velocity was 3.9 m/s (*Panel B*). A decision was made to attempt to resolve the thrombus with repeated intravenous infusions of alteplase.^1,2^ Four sequential infusions of 25 mg alteplase/25 h resulted in an uneventful but stepwise decrease of the transvalvular peak velocity to 2.8 m/s (*Panel C–F*). Multislice computed tomography confirmed normalization of leaflet motion with some residual remaining leaflet thickening (*Panel F*). MSCT cine images before and after alteplase infusion are available in the Supplementary data online (*Videos S1* and *S2*).

**Figure 1:**
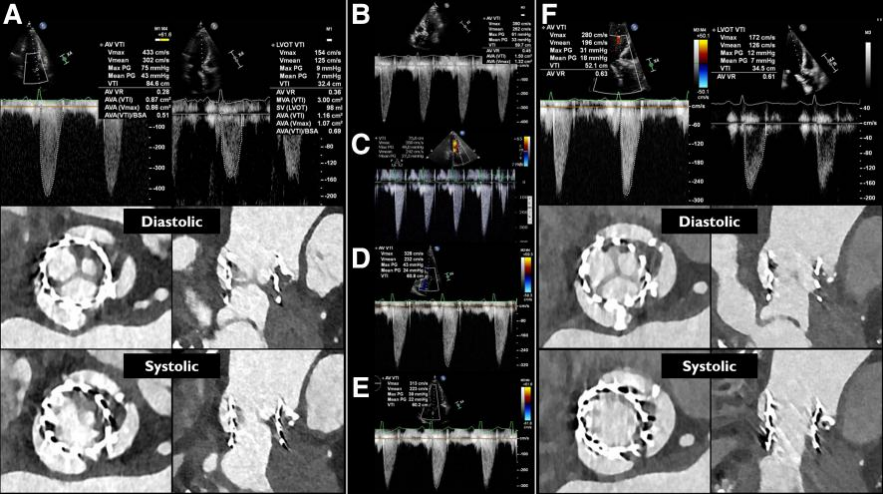


Clinical valve thrombosis is relatively uncommon after TAVI but has serious clinical implications including a risk for thrombo-embolic events and heart failure.^3,4^ Intravenous low-dose thrombolytic therapy may resolve clinically significant valve thrombosis after TAVI when oral anticoagulant regimens have failed.

##  

Supplementary data is available at *European Heart Journal* online.

No data were generated or analysed for this manuscript.

R.A. has nothing to declare. R.P.J.B. has received institutional support by Siemens. N.M.V.M. has received grant support by Abbott, Boston Scientific, Biotronic, Edwards Lifesciences, Medtronic, PulseCath BV, Abiomed, and Daiichi Sankyo; consulting fees by Jenavalve, Daiichi Sankyo, Abbott, Boston Scientific, and Medtronic; payment or honoraria for lectures, presentations, speakers, manuscripts, and educational events by Abiomed and Amgen; and support for attending meetings by Jenavalve.

## Supplementary Material

ehad303_Supplementary_Data
